# Age-friendly communities and well-being realization among older native and immigrant populations in the Netherlands: a theory-guided study protocol

**DOI:** 10.1186/s12877-022-02880-4

**Published:** 2022-04-02

**Authors:** Anna P. Nieboer, Jane M. Cramm

**Affiliations:** Erasmus School of Health Policy & Management, Erasmus University Rotterdam, P.O. Box 1738, 3000 DR Rotterdam, the Netherlands

**Keywords:** Older people, Immigrant, Theoretical model, Mixed-methods, Age-friendly communities, Well-being, Solidarity, Study protocol

## Abstract

**Background:**

With rapid population aging, policy makers and service providers are becoming increasingly aware of the importance of building and maintaining age-friendly communities. Clearly, “age-friendly” relates to the impact of context on people’s well-being. But how? What is an age-friendly community, and does that differ for native and immigrant older people? Up until now, how native and immigrant older people in the Netherlands perceive community age-friendliness, and whether and how age-friendly communities help them realize well-being, remains unknown which limits opportunities to develop appropriate interventions. This article presents a study protocol to identify, theoretically and empirically, how and under what conditions age-friendly communities help native and immigrant older people in the Netherlands realize well-being.

We present a theory-guided approach to elucidate differences in neighborhood age-friendliness and requirements for age-friendly community development between native Dutch and immigrant older people. Good interventions are built on good theory. The proposed research will add to theory building by systematically examining what older people get from their neighborhoods and the conditions that influence well-being realization, including the role of individual and neighborhood resources. We posit that physical and social well-being realization will be enhanced in age-friendly communities that support realization of multiple well-being needs and development of solidarity within and between groups in the neighborhood via cross-cutting sharing arrangements.

**Methods:**

We present a mixed-methods design among native and immigrant older people (Turkish, Surinamese and Moroccan) consisting of: (i) Q-studies (combining in-depth interview-based and quantitative analyses); (ii) a pilot survey study; (iii) a main survey study in Rotterdam, the Hague, Utrecht, and Amsterdam; and (iv) focus groups.

**Discussion:**

By exploring truly new ground in the field of age-friendly communities, the results of the proposed research will provide new empirical evidence, advance theory, and be helpful for the development of interventions aimed at improving age-friendliness and well-being for native and immigrant older populations, thereby contributing to resolving the societal challenges of caring for and supporting older people in the community.

## Background

With populations aging rapidly, service providers and policy makers are increasingly aware of the importance of building and maintaining age-friendly communities [1]. Clearly, “age-friendly” relates to the impact of context on people’s well-being. But how? What is an age-friendly community, and does that differ for native and immigrant older people? Up until now, how native and immigrant older people in the Netherlands perceive community age-friendliness, and whether and how age-friendly communities help them realize well-being, remains unknown. People’s need for neighborhood resources appears to increase as their individual resources decline with age [2]. Although research shows that age-friendly environments within the neighborhood positively affect older people’s well-being [2, 3], theory about why some neighborhoods are more age-friendly than others and how such age-friendly communities are related to older people’s well-being is lacking [1], especially for older migrants. The bottom-up approach the WHO followed in the development of their age-friendly cities guide yielded a rather idiosyncratic list of neighborhood resources [4]. Moreover, even if we would know which neighborhood resources are relevant, we lack validated measurement instruments to assess these resources and community age-friendliness for migrant populations. It is unknown how native Dutch and immigrant older people perceive community age-friendliness, and whether and how age-friendliness helps them realize well-being. The literature on aging in place lacks attention to ethnicity. This article presents a study protocol to identify, theoretically and empirically, how and under what conditions age-friendly communities help native and immigrant older people in the Netherlands realize well-being.

### A theoretical model to study how and under what conditions age-friendly communities help native and immigrant older people in the Netherlands realize well-being

**Fig. 1:**
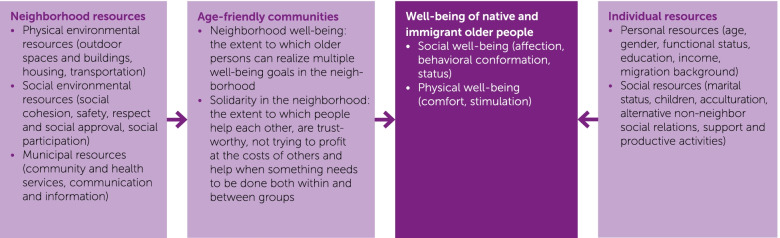
Theoretical model: Neighborhood and individual resources, age-friendly communities and well-being realization

To elucidate differences in neighborhood age-friendliness and requirements for age-friendly community development between native Dutch and immigrant older people a theory-guided approach [5] will be used (see Fig. 1 for a theoretical model overview). This research will add to theory building by systematically examining what older people get from their neighborhoods and the conditions under which well-being realization is more (or less) likely. What is an age-friendly community and does that differ for native and immigrant older people? To gain a systematic understanding of how people benefit from a community, we need a theory of well-being needs and substitutable resources [6, 7]. Social production function (SPF) theory [8, 9] provides a full characterization of people’s ability to achieve well-being. According to SPF theory, need satisfaction is best viewed in terms of production functions. That is, a particular level of need satisfaction (output) is “produced” by a particular input, wherein people are producers of their own well-being by way of need fulfilment [9].

### Well-being of native and immigrant older people

Lindenberg [8] identified five substantive needs (or goals) which must be fulfilled to achieve social and physical well-being: behavioral confirmation, affection, status, stimulation and comfort. Social well-being is achieved by: (i) living according to certain values and norms (behavioral confirmation); (ii) receiving enough affection through friendship, intimacy, and emotional support and; (iii) having a certain status based on one’s occupation, lifestyle, or talents. Physical well-being can be achieved by: (i) being in a situation of optimal comfort; and (ii) creating proper amounts of mental and physical stimulation [9, 10]. In the SPF theory hierarchy, the substantive needs fall below two ultimate needs (social and physical well-being) and above resource-related needs required for their production (e.g., income, health care, social network) [9, 11]. Production functions specify factors needed to fulfil a need. An array of production functions relating needs across levels can show how well-being is generated, maintained, and changed.

Highly efficient activities and resources are multifunctional; they serve more than one need and contribute to short- and long-term well-being. Substitution may occur based on needs’ relative costs: one may intensify social interactions (affection, behavioral confirmation) when opportunities to gain status (e.g., volunteer work) decrease. People may pursue resources for higher-level needs, despite declining returns, thereby increasing buffering reserves to draw from [12]. This needs hierarchy is useful for determining how well-being is achieved across populations [13] and for identifying the types of care and support required at individual and neighborhood levels to overcome obstacles to achieving well-being.

### Age-friendly community

We may speak of community when community-dwelling older people realize multiple well-being needs together [13]. A community can thus be seen as a collection of multifunctional relationships conditioned by membership benefits, together with opportunities for and ease of need realization [14]. If people create communities expecting well-being realization [13, 14], then the investigation of relationships between neighborhood characteristics as conditions under which this realization is more (or less) likely is a promising approach to identify the importance of age-friendly communities for the well-being of older people [13]. Lindenberg’s theory of community [6, 13] assumes that communities enable individuals to realize well-being needs via joint production. People depend on others for affection, behavioral confirmation, status, comfort, and stimulation. The extent to which older people can realize these substantive well-being needs in a neighborhood underlie, in part, a community’s age-friendliness.

While dealing with aging, declining mobility [15], and reliance on smaller social networks [16], living in an environment with solidarity among neighbors may enhance well-being as well. Solidarity refers to people helping each other when there is a need for it (even when inconvenient), people are trusting and trustworthy, not trying to profit at the costs of others, and everyone helps when something needs to be done [17]. Such a neighborhood could be an important social resource, especially in times of de-institutionalization and increasing self-responsibility, to realize the goals that lead to physical and social well-being. Solidarity among community members influences the realization of affection and status through the provision of affective support and enhancement of self-esteem and mutual respect. Neighbors taking caring and looking out for one another may improve older people’s comfort levels [18]. Such solidarity might attenuate the adverse effects on need fulfillment caused by aging-associated losses and challenges [19]. In contrast, poor neighbor solidarity may augment stress due to need realization deficits [20].

The call for neighbor solidarity in support of aging in place should not neglect how strong solidarity among tight-knit groups leads to boundaries that engender exclusionary, hostile behavior vis-a-vis outgroups [17]. Little is known about the production of solidarity in interaction among and between native and immigrant older people in neighborhoods. Urbanization and living in a neighborhood with migrants can disrupt solidarity [14]. Strong solidary relations between migrants may augment well-being within the group, but they may not enhance solidarity between groups within the same neighborhood. Consistent with Putnam’s [21] hypothesis that solidarity is lower in ethnically diverse neighborhoods, Gijsberts and colleagues [22] reported that ethnic diversity can reduce contact in neighborhoods (an indicator of social cohesion and prerequisite of community), but they found no effects on trust in others, volunteer work, and informal support provisions. Glas and colleagues [23] however did report a diversity effect on neighborhood cohesion.

Sharing group theory helps us understand interdependencies among people and the conditions that lead to cooperative arrangements [6, 24]. If people in a neighborhood have to share goods and if they have to make arrangements concerning the use of goods (e.g., the street they live in, parking lots, trash cans), they establish contact with one another and -sometimes as a byproduct- solidary relations and community emerge [14]. Being dependent on solidary relationships in the neighborhood could however also result in unwanted intrusions into one’s privacy or a high degree of social control [7, 14], which compromise affection and behavioral confirmation. Sharing a neighborhood may produce both positive (behavioral conformation for “doing the right thing”) and negative externalities (e.g., discomfort due to neighbors’ nuisance behavior). If little is shared, social norms will be vague, information costs of what is right versus wrong in informal interactions high, and behavioral confirmation need fulfilment not a by-product of daily living. Moreover, activities by which one realizes affection and behavioral confirmation can become clustered by ethnicity and other characteristics, such as age and education [14], and may reduce cross-cutting sharing arrangements that create overlapping sharing groups (e.g., having family and friends that are also neighbors, being with neighbors while doing volunteer work, or meeting neighbors at a Mosque). Lack of overlapping sharing groups of native and immigrant older people in neighborhoods will limit solidarity between these groups and reduce opportunities for well-being realization.

We posit that physical and social well-being realization will be enhanced in age-friendly communities that support realization of multiple well-being needs and development of solidarity within and between groups in the neighborhood via cross-cutting sharing arrangements. Our theoretical model explains under what conditions older people generally realize well-being (Fig. 1). First, neighborhood resources that influence age-friendly community development—including physical-environmental, social-environmental, and municipal resources—are outlined. Both the realization of multiple well-being needs and solidarity in the neighborhood are constituents of an age-friendly community. Age-friendly communities together with individual resources in turn explain native and immigrant older people’s well-being.

In addition to identifying the conditions under which native and immigrant older people realize well-being generally, we need further theorizing about how to create cross-cutting sharing groups and understand differences in well-being realization between native and immigrant older people. Diversity in individuals’ resources, life-courses, and contextual factors underlie such differences [13, 25], especially between natives and immigrants [26]. Non-Western older immigrants in the Netherlands are concentrated within disadvantaged neighborhoods in large cities [27], representing a double burden [28]. Living in a disadvantaged neighborhood has detrimental socio-economic consequences, including crime, poverty, substandard housing, overcrowding, and noise pollution. Immigrants' tendency to stay in their original homes indicates they are even further lagging behind in the housing market as compared with natives. Differences are also found across immigrant groups: Surinamese older people are often in better housing and neighborhood conditions than Turkish and Moroccan older people in the Netherlands [29]. Regarding social-environmental resources, living in immigrant enclaves can contribute to the secondary burden of living with social inequality and discrimination [30]. Conversely, most older migrants are embedded in social relationships that provide affection, and they are respected highly in their communities, which enhances status realization. The high status of older people in the Muslim social hierarchy may support realization of social well-being needs in a way not seen among elder natives. However, if older Muslim immigrants lack social bonds within their own community they are especially vulnerable. Overall, older immigrants contribute to civil society through fulfilling important roles and providing support to their families, neighbors, and local community [31], which helps them realize status, behavioral confirmation, and stimulation needs. While language barriers can prevent immigrants from connecting with people outside their community, migrants often have strong social bonds with people who share the same ethnicity and background, which can support solidarity while, simultaneously, impeding cross-cutting among sharing groups of native and migrant groups [31]. This observation is in line with natives who also prefer to live among people with similar (non-immigrant) backgrounds [25] and raises questions regarding whether heterogeneity should be avoided or not. If not, what can make heterogeneity contribute to age-friendly communities in such a way that conflict and subsequent parochial sharing groups are avoided? The leading hypothesis of the study is if neighborhood resources enhance the age-friendliness of a community (i.e., increase neighborhood well-being realization and solidarity within and between groups), then, ceteris paribus, well-being realization will be higher and differences between natives and migrants smaller.

We aim to further develop theories by explicitly and systematically formulating expected relationships among age-friendly community and well-being realization, and the conditions under which they are expected for native and migrant populations so they can be used to guide future research and intervention development. We will also apply our theory-guided approach to further instrument development for migrant populations. Previously [3, 5, 25], we put much effort into refining the conceptualization of well-being realization, neighborhood resources, and age-friendly community, as suggested by Lindenberg’s SPF theory and theory of community, and into developing reliable measures for these concepts, but this hasn’t been done yet for natives as well as immigrant populations.

## Methods

The study aim to identify, theoretically and empirically, how and under what conditions age-friendly communities help native and immigrant older people in the Netherlands realize well-being will be achieved through four objectives:

1. To identify views among native and immigrant older people on the importance of neighborhood resources for age-friendly communities and how they aid well-being realization.
2. To develop and validate measurement instruments to assess neighborhood resources and age-friendly community among native and immigrant older people.
3. To firstly describe the level of well-being realization, age-friendly communities and individual and neighborhood resources of native and immigrant older people, and secondly explain differences in well-being realization among native and immigrant older people.
4. To identify practical implications, make policy recommendations and develop interventions for the fostering of age-friendly communities in the neighborhood for native and immigrant older people.

**Fig. 2:**
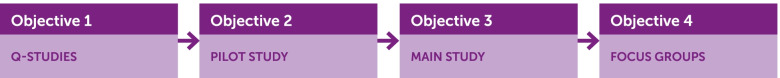
Work plan

A mixed-methods design will be used to address these objectives (Fig. 2) [32]. The project is empirically and theoretically innovative in that it is the first to investigate requirements for age-friendly community development for native and immigrant older people, and how these communities support their well-being realization, using a unique mixed-methods design.

### Objective 1: to identify views among native and immigrant older people on the importance of neighborhood resources for age-friendly communities and how they aid well-being realization

In line with our theoretical model, we will explore prevailing perspectives on the importance of neighborhood resources for age-friendly communities and how they contribute to well-being realization. We will conduct four Q-studies (combined in-depth interview-based and quantitative analyses) with four groups of older people (*N* = 20/group): natives, Turkish, Moroccan, and Surinamese immigrants. Q-methodology is designed to explore respondents’ subjective perspectives [33]. Respondents will be presented with sample opinion statements about neighborhood resources and instructed to rank them according to their agreement with how each would support well-being realization. Additionally, qualitative material will be collected by asking respondents to explain their statement rankings and answer follow-up questions. We will identify significant clusters of correlations among rankings through by-person factor analysis. An assumption underlying this analysis is that respondents who ranked the statements similarly will have similar perspectives on neighborhood resources and how they support well-being realization. For each factor, a composite ranking of the statements will be computed and then serve as the basis for interpretation and description of the factor as a perspective on neighborhood resources for age-friendliness and how they support well-being realization, as in [25].

### Objective 2: to develop and validate measurement instruments to assess neighborhood resources and age-friendly community among native and immigrant older people

Building on our previously developed and validated well-being instrument for native and immigrant older people [5], this project will deliver validated instruments for the assessment of neighborhood resources and community age-friendliness among Dutch, Moroccan, Turkish, and Surinamese older people, enabling comparison among these groups. A pilot survey will be conducted with these populations (*N* = 200 each) for instrument validation. The following steps will be used for development and validation purposes. First, the *neighborhood resources* assessment will be further developed and validated with these populations building on previous work [2, 13] in which aging in place was assessed based on the World Health Organization (WHO) framework for age-friendly cities and additional related literature [1, 25]. This instrument measures eight WHO-defined domains: social participation; transportation; outdoor spaces and buildings; housing; civic participation; communication and information; respect and social approval; and community support and health services. We will develop an instrument to assess the full breadth of neighborhood resources (physical environmental resources, social environmental resources, and municipal resources) for these populations based on earlier work [25], including our studies on social cohesion and neighborhood services [34, 35]. Second, the *age-friendly community* instrument will be built upon the work of Völker and colleagues [7], who assessed the extent to which community dwelling older people realize multiple well-being goals together in the neighborhood. It will incorporate questions on the realization of affection, behavioral confirmation, status, comfort, and stimulation in the neighborhood. Third, *solidarity in the neighborhood* will be measured with an instrument from our earlier work [3]. This scale was based on Lindenberg’s theory of solidarity, in which group members contribute to collective success, are prepared to help others in need, resist the temptation to let other members do most of the work, share responsibilities, and are prepared to apologize for mistakes [17]. It was modified to assess solidarity within a neighborhood [3].

Before the quantitative pilot study, statement comprehensiveness and unambiguity of these instruments will be tested during interviews. The instruments will be translated to respondents’ native languages. Data will be screened for missing values and item means and standard deviations. Confirmatory factor analyses will be used to test instrument validity in LISREL with Hu and Bentler’s cut-off criteria [36]. Multigroup analyses will be carried out across population groups.

### Objective 3: to firstly describe the level of well-being realization, age-friendly communities and individual and neighborhood resources of native and immigrant older people, and secondly explain differences in well-being realization among native and immigrant older people

To enroll ~ 3,000 people for the main survey, a representative sample of 7,500 community-dwelling older people (≥ 65 years) in the Hague, Rotterdam, Amsterdam, and Utrecht will be identified from the population registrar for inclusion. A two-stage design will be used in which neighborhoods will be sampled first and then individuals will be selected to be proportionate to neighborhoods according to age (65–69, 70–74, 75–79, 80–84, 85 + years) and migration background. Eligible older people will be asked by mail to complete a written or online questionnaire. Reminders will be sent, first by mail and next by telephone. If they cannot be reached, non-respondents will be visited at home by interviewers with the same cultural background. The written invitation, questionnaire, and reminder will be provided in Turkish, Arabic, and Dutch. Prior to participation, each respondent will be informed of the aims of the study and its anonymous and voluntary nature. We used this strategy in previous research and expect a 40% (*N* = 3,000) response rate.

The theoretical model (Fig. 1) provides the starting point for assessment of neighborhood resources and age-friendly communities (both neighborhood well-being and solidarity in the neighborhood), as well as the individual resources and well-being of native and immigrant older people. Neighborhood resources and community age-friendliness will be assessed with the validated measurement instruments developed in the pilot study. The instruments for assessment of individual resources and well-being are described below.

*Well-being* will be measured with the 15-item Social Production Function Instrument for Level of Well-being (SPF-IL) [5, 37], which measures physical (comfort, stimulation) and social (behavioral confirmation, affection, status) well-being levels. The SPF-IL is a reliable instrument to assess well-being in native and immigrant older populations [5].

*Individual resources* refer to personal resources (age, sex, functional status, education, income, and migration background) and social resources (marital status, children, acculturation, alternative non-neighbor social relations, support, and productive activities). Well-known instruments will be used to assess these variables (e.g., functional status will be measured with the Short Form 20 Health Survey, acculturation with the Psychological Acculturation Scale [38, 39], social support with the Oslo social support scale (OSSS-3) [40] and (limitations in) productive activities with the Activity Restriction Scale [41]. No licenses are needed for these instruments.

(Existing) data registries will be used for data describing variation in neighborhood heterogeneity, social inequality, social security, safety, nuisance and neighborhood SES. We will use the Herfindahl–Hirschman-Index (HHI) and relative proportions of the three ethnic groups in each neighborhood to measure ethnic diversity. This index represents the probability that two randomly selected individuals will hail from different ethnic groups. The higher the index value, the more ethnic groups are present, and the less the neighborhood is dominated by native Dutch [22]. In addition, we will also use a modified HHI measure (a more innovative method to measure the ethnic composition of neighborhoods) which is group-specific and assesses the level of diversity among members of the out-group (the group a certain person does not belong to) from the perspective of the in-group (the group a certain person does belong to) [23].

We will estimate hierarchical random-effects models that account for the nested nature of the data to assess relationships between well-being, individual characteristics, neighborhood resources, and age-friendly communities as well as between-group and within-group differences. Moderated mediation analyses will be performed to assess (1) whether the well-being impact of particular neighborhood characteristics differs between older migrants and natives, and (2) the extent to which differences in neighborhood resources explain well-being differences between older migrants and natives. The bootstrapping procedure proposed by Preacher and Hayes in 2008 [42] will be used to estimate standard errors. The proposed sample size (*N* = 3,000) is considerably larger than the sample size of earlier data collections among older people with a migration background in the Netherlands (e.g., Longitudinal Aging Study Amsterdam (LASA; *N* = 478), Gezondheid en welzijn van allochtone ouderen (GWAO; *N* = 1,811). This reflects the statistical power requirements of the proposed analyses.

### Objective 4: to identify practical implications, make policy recommendations and develop interventions for the fostering of age-friendly communities in the neighborhood for native and immigrant older people

Seven focus groups (5–7 participants each) will be held with natives (group 1), with Turkish (group 2), Moroccan (group 3), and Surinamese (group 4) immigrants, and with policy makers (groups 5–7). The findings from objectives 1–3 will be discussed as well as practical implications, policy recommendations, and input to develop interventions and a webtool. Participants will be asked to help translate project results, practice implications, and policy recommendations into interventions and a web-tool that will help municipalities identify changes that will enable aging in place and realization of well-being. This approach and design builds on our earlier work in which we developed a tool for municipalities to improve integrated care in the community (see https://www.integraalwerken.nl/), which has been used widely (e.g., Rotterdam, Breda, Elburg, Bergen op Zoom). This tool was developed in collaboration with policy makers and native older people. We will build on this work to develop interventions and a webtool for municipalities to assess age-friendliness and identify which resources contribute to an age-friendly community and well-being. In the final project phase, we will approach municipalities for a follow-up study to test the interventions and webtool thus ensuring continuation and practical dissemination of our results.

## Discussion

An environment supportive of aging in place is characterized by living conditions that are safe, comfortable, pleasant, and stimulating, thus enabling people to fulfill their well-being needs. To achieve this, the neighborhood in which people live should be characterized by personal and longstanding relationships. Relationships in which people really make you feel that you can count on one another and not just helping each other in practical ways. It is a circle of mutual relationships, not in the sense of a simple ‘tit for tat’, but rather a willingness to pay attention to one another and helping those in need. It is a matter of mutual solidarity in the neighborhood and the creation of an age-friendly community [3]. Moreover, strengthening social relationships in an age-friendly community is expected to be the way forward in lowering socio-economic (health) inequalities [43]. A society lacking such social relationships is characterized by conflict, disparate moral values, social disorder, social inequality, little attachment to place, and low levels of social interaction among and within communities. Although local interactions within communities have declined in the past decades, it continues to be important for many people [7]. However, beyond-local ties are increasing and becoming more dissociated from forms of local interaction with fewer overlapping sharing groups [44]. People are socializing within and outside the neighborhood, but these activities are different from one another. Older people and people outside the labor force show little change in neighboring patterns and are apparently more dependent on local ties [45]. There are many neighborhoods where people have relatively low neighborhood engagement. In such neighborhoods, people may nonetheless enjoy a high degree of livability because they are not nuisances to one another, they feel safe, and each individual looks after the neighborhood aesthetic [44]. However, when neighborhood livability becomes an issue, such as in disadvantaged neighborhoods with large immigrant populations, collaboration among people is often a necessity for improvement. In neighborhoods dealing with livability issues, widespread collaboration among people does not come about naturally and its longevity cannot be taken for granted when it does arise. Neighborhoods dealing with those disadvantages tend to lack the qualities of self-organization, trust, and mutuality that would aid their regeneration, explaining, in part, the cumulative outcomes of their decline [46]. Knowing how and under which conditions neighborhood communities facilitate well-being realization for natives as well as immigrants will have scientific and societal impacts.

### Strengths and limitations

A major strength of the study is that we aim to further develop theories by explicitly and systematically formulating expected relationships among age-friendly community and well-being realization, and the conditions under which they are expected for native and migrant populations so they can be used to guide future research and intervention development. We will also apply our theory-guided approach to further instrument development for migrant populations. Previously [3, 5, 25], we put much effort into refining the conceptualization of well-being realization, neighborhood resources, and age-friendly community, as suggested by Lindenberg’s SPF theory and theory of community, and into developing reliable measures for these concepts, but this hasn’t been done yet for natives as well as immigrant populations. We use a unique mixed-methods design with a combination of quantitative and qualitative research methods to address requirements for age-friendly community development for native and immigrant older people, and how these communities support their well-being realization.

The proposed study has potential limitations and challenges, however. First, the response rate is known to be lower among Turkish and Moroccan older people than for natives [47, 48]. To improve response rates, we aim to have at least ten contact attempts given that previous research shows that a minimum of six contact attempts is needed for Turkish and Moroccan samples [48]. In addition, in the pilot study we will vary with incentives; 5 euros beforehand (regardless of people filling in the questionnaire or not), 5 euros after filling in the questionnaire, 5 euros beforehand and 5 euros after filling in the questionnaire, and ten euros afterwards. Based on the effectiveness of these incentives we will choose the most effective incentive per group for the main study. Second, the covid-19 crisis is still ongoing, which is expected to influence our study findings. This however also represents a unique moment in time to collect data about ageing in place among natives and immigrant populations. Especially, given that during the COVID-19 pandemic the dependence on the neighborhood and neighborhood resources became stronger [49]. As movements were often restricted both nationally and internationally, people were confined to their home and immediate neighborhood [50]. Furthermore, we know that people with a migration background are hit even harder by the COVID-19 crisis compared to people without a migration background [51–53]. This research will therefore lead to unique knowledge on ageing in place during COVID-19 times. The proposed research will provide insight into well-being realization and age-friendliness of communities in the Netherlands and inform policy recommendations for aging in place. Comparable studies are needed in other countries, with other neighborhood resources and solidarity levels to compare our findings. The focus on how neighborhoods can support aging in place and well-being is timely also outside the Netherlands given the call for more community support for older people. The same picture emerges in other Western countries where governments are implementing policies fostering active citizenship and making citizens self-reliant as long as possible. With these policies, neighborhoods and local communities may become critical for enabling older people to age in place [29]. The stay-at-home ordinances enacted in response to the COVID-19 pandemic accentuated peoples’ dependence on neighborhood resources (e.g., regarding neighborly help, public assistance programs, housing, health care, community care). The proposed research is responsive to the realization that de-institutionalization and increasing self-reliance leads to greater dependence on neighborhood resources and the lack of data regarding the needs of migrant older people hoping to age in place successfully and how they might differ from native older people’s needs.

## Data Availability

This project will involve data from respondents with and without a migration background in the Netherlands. These data will be shared 1 year after completion of the project. Surveys and focus group discussion guides have not been developed yet, but will also be available during and/or after the study. Those who are interested in obtaining the data and/or other material can contact Professor Anna Petra Nieboer.

## Abbreviations

HHI: Herfindahl-Hirschman-Index
SPF-IL: Social Production Function Instrument for the Level of well-being
SES: Socio-economic status
